# Left-Sided Sensorineural Hearing Loss and Facial Weakness in a 35-Year-Old Patient: A Diagnostic Challenge and Case Report

**DOI:** 10.7759/cureus.41606

**Published:** 2023-07-09

**Authors:** Osmay Cardoso, Mohamad Hamade, Khushi Saigal, Richard Wang, Gaurav Saigal

**Affiliations:** 1 Radiology, University of Miami Miller School of Medicine, Jackson Memorial Hospital, Miami, USA; 2 Radiology, University of Florida College of Medicine, Gainesville, USA; 3 Radiology, University of Miami, Miami, USA

**Keywords:** neurologic sarcoidosis, igg4 disease, cns lymphoma, s: magnetic resonance imaging, diagnostic reasoning, s: vestibular schwannoma, sudden sensorineural hearing loss (ssnhl)

## Abstract

We present the case of a 35-year-old patient who presented with a three-month history of left-sided sensorineural hearing loss and left-sided facial weakness. Initial imaging suggested a schwannoma, and the patient underwent ten treatments of intra-tympanic steroid injections and antibiotics, and was scheduled for surgery. However, the planned schwannoma removal surgery with gamma-knife was aborted due to the absence of the previously identified mass on the pre-procedure MRI. Subsequent imaging revealed continued enhancement of the left internal auditory canal (IAC), leading to considerations of lymphoma, sarcoidosis, IgG4 disease, or other inflammatory condition. The patient's symptoms have significantly improved since and are currently being conservatively managed and monitored. However, the patient continues to show persistent findings on MRI. This case highlights the diagnostic challenges faced in identifying the underlying etiology of this patient and emphasizes the need for further investigations and multidisciplinary management in patients with similar presentations.

## Introduction

Sensorineural hearing loss and facial weakness are concerning symptoms that can be caused by various etiologies, including neoplastic, inflammatory, and infectious conditions [1,2]. Differentiating between these causes is crucial for appropriate management and prognostication. [2] We describe a case of a 35-year-old patient who presented with a three-month history of left-sided sensorineural hearing loss and left-sided facial weakness. The initial suspicion of schwannoma was challenged by subsequent imaging findings. The loss of the initial mass-like enhancement on MRI coupled with the continued enhancement of the geniculate ganglion, tympanic, and mastoid segments of the facial nerve raised concerns for lymphoma, sarcoidosis, IgG4 disease, or another inflammatory condition.

## Case presentation

**Figure 1:**
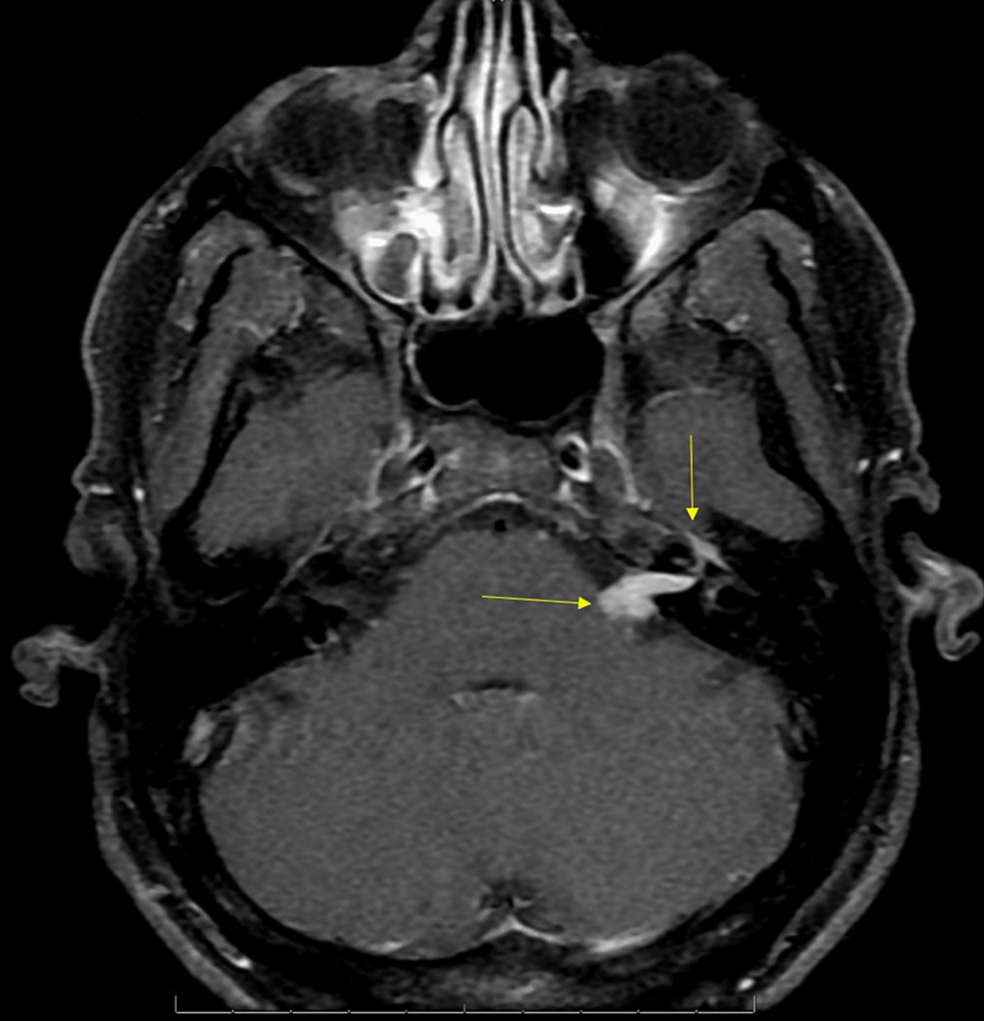
Initial axial T1 fat-saturated sequence MRI of the brain The image shows a mass-like enhancement of the left internal auditory canal, geniculate ganglion, and left seventh cranial nerve (seen by arrows above) consistent with the initial diagnosis of schwannoma.

**Figure 2:**
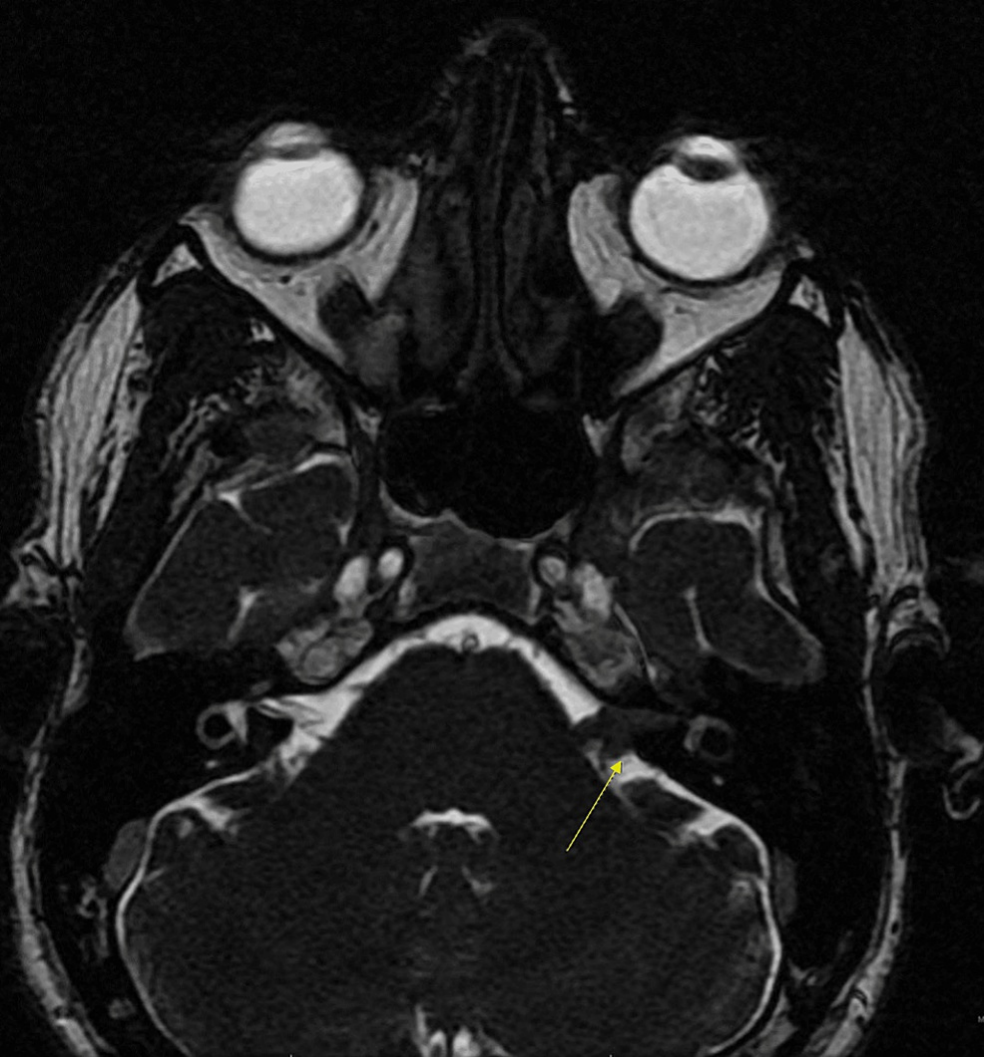
Initial axial T2 heavy-weighted (FIESTA/CISS) sequence MRI of the brain The image shows an abnormally decreased signal in the left internal auditory canal and inner ear structures (arrow). CISS - Constructive Interference Steady State

A 35-year-old previously healthy male presented with a three-month history of progressive left-sided sensorineural hearing loss and left-sided facial weakness. Physical examination revealed profound left sensorineural hearing loss and a left peripheral lower-motor neuron facial nerve paralysis, which was deemed grade IV on the House-Brackmann scale. Otoscopy findings were unremarkable. An initial T1 sequence MRI showed a mass-like enhancement in the left internal auditory canal, geniculate ganglion, and left seventh cranial nerve consistent with a schwannoma (Figure 1). The patient's initial T2 - heavy weighted (FIESTA/Constructive Interference Steady State (CISS)) sequence MRI of the brain showed decreased signal in the same region as seen in (Figure 2).

Management and follow-up

**Figure 3:**
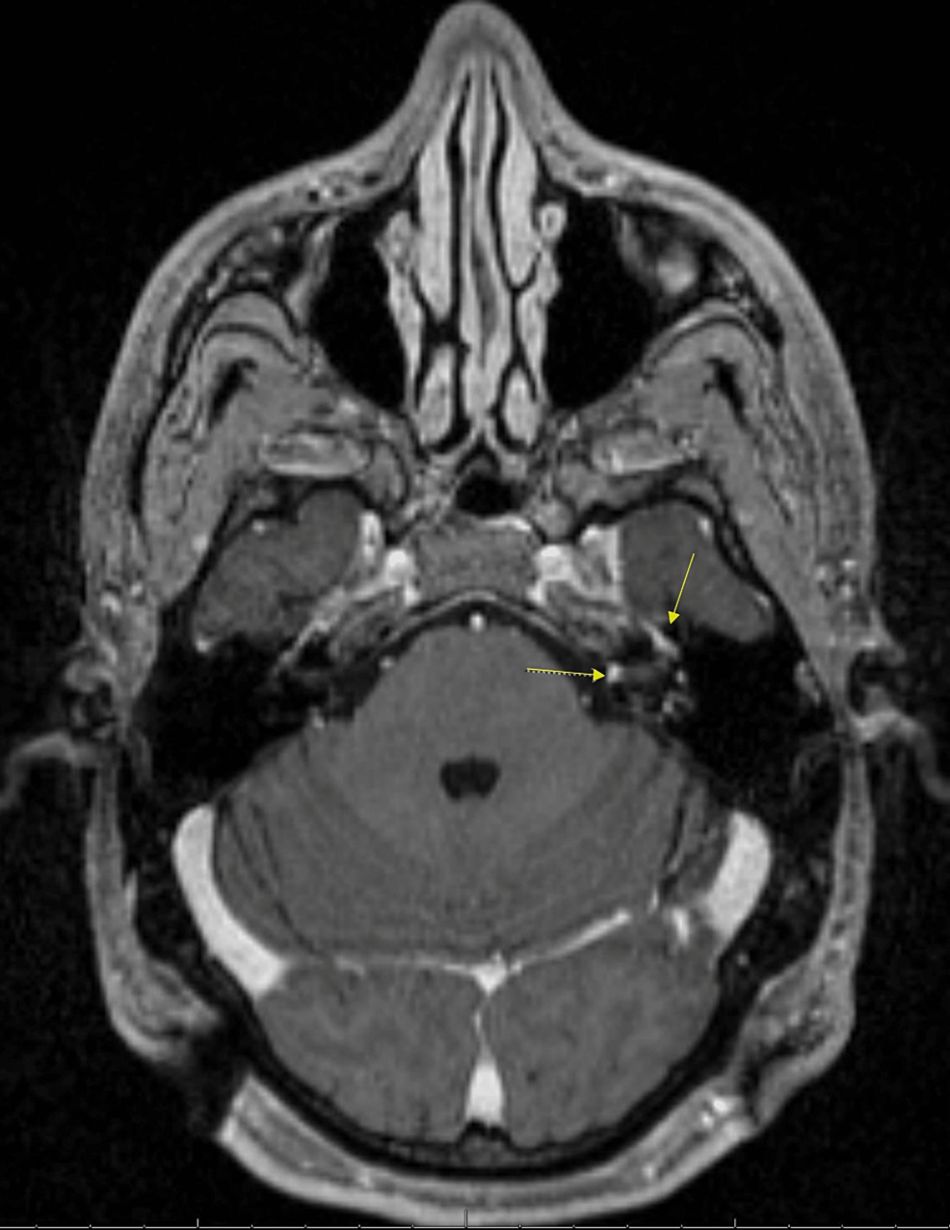
Axial T1 contrast MP RAGE sequence MRI of the brain, pre-gamma knife procedure four months after initial imaging The image shows residual enhancement in the left porus acusticus as well as the left apex of the cochlea shown by the arrows above.

**Figure 4:**
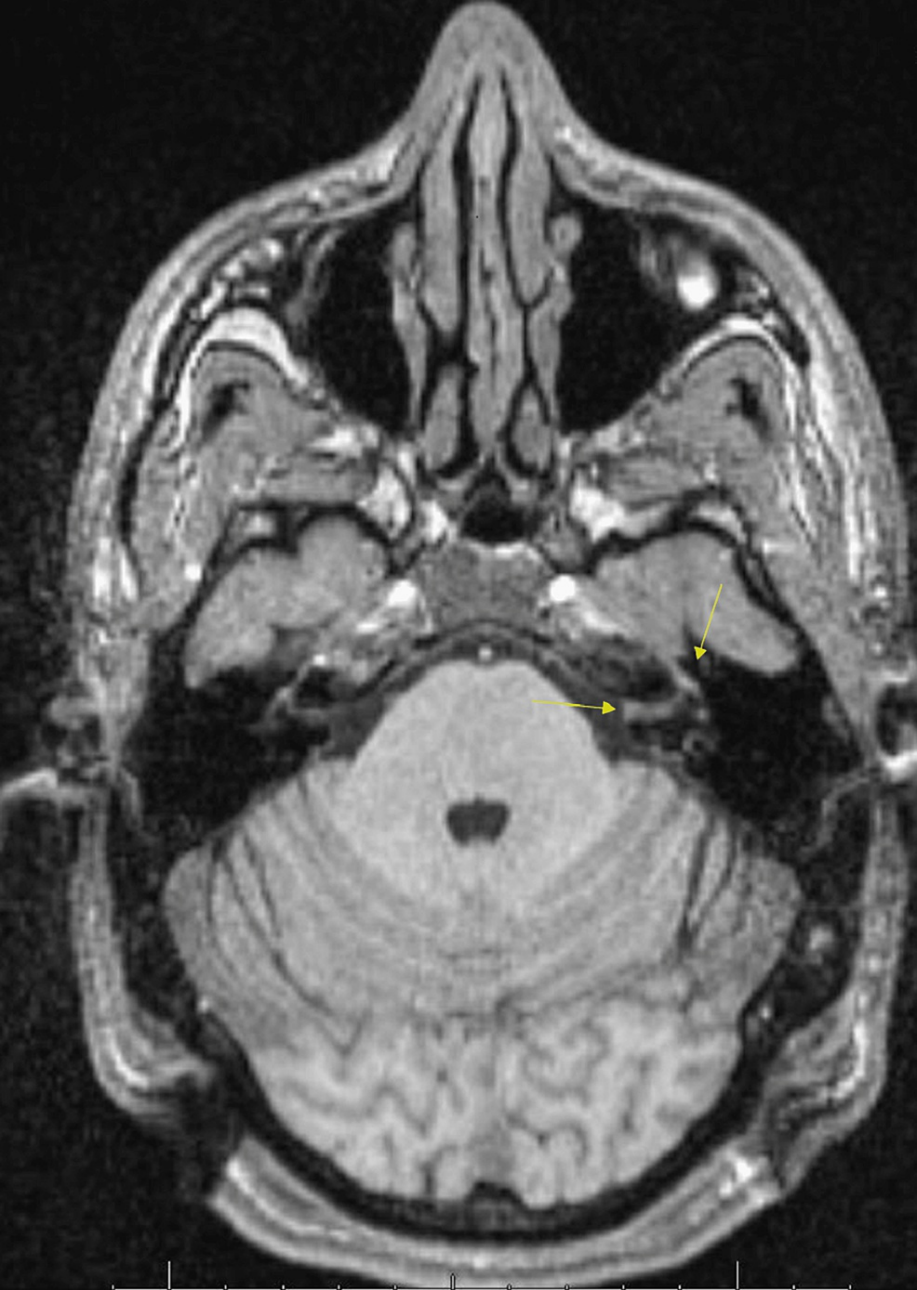
Axial T1 pre-contrast MP RAGE sequence MRI of the brain, pre-gamma knife procedure four months after initial imaging The image shows bright regions in the left porus acusticus as well as the left apex of the cochlea seen in the arrows above.

The patient received a course of 10 intra-tympanic steroid injections alongside antibiotics and was scheduled for a gamma-knife removal surgery four months after the presentation. The treatment aimed to reduce inflammation and manage any potential concurrent infection prior to the procedure. The patient remained symptomatic, with minimal improvement during this time. The pre-procedure pre- and post-contrast MP RAGE sequence MRI revealed the absence of the previously identified mass, showing only residual enhancement (Figures 3, 4). This perplexed the surgical team and raised suspicion for alternative etiologies, including lymphoma, sarcoidosis, IgG4 disease, or another serious inflammatory condition. The gamma-knife procedure was ultimately aborted.

**Figure 5:**
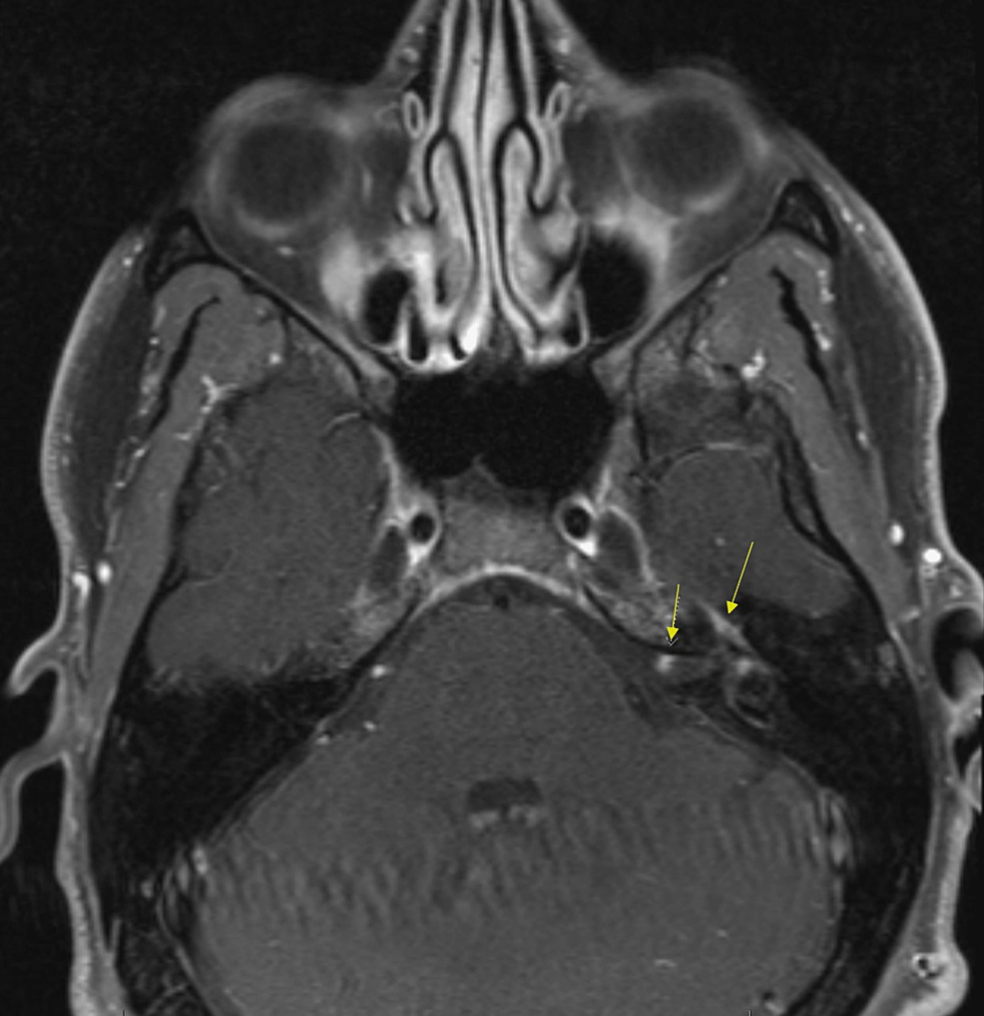
Axial T1 fat-saturated sequence post-contrast MRI of the brain seven months after initial imaging The image shows decreased enhancement of the left internal auditory canal (IAC) and stable asymmetric enhancement of the left geniculate ganglion, tympanic segment, and descending mastoid segment of cranial nerve seven, apical turn of the cochlea, vestibule, and lateral and posterior semicircular canal (arrows).

**Figure 6:**
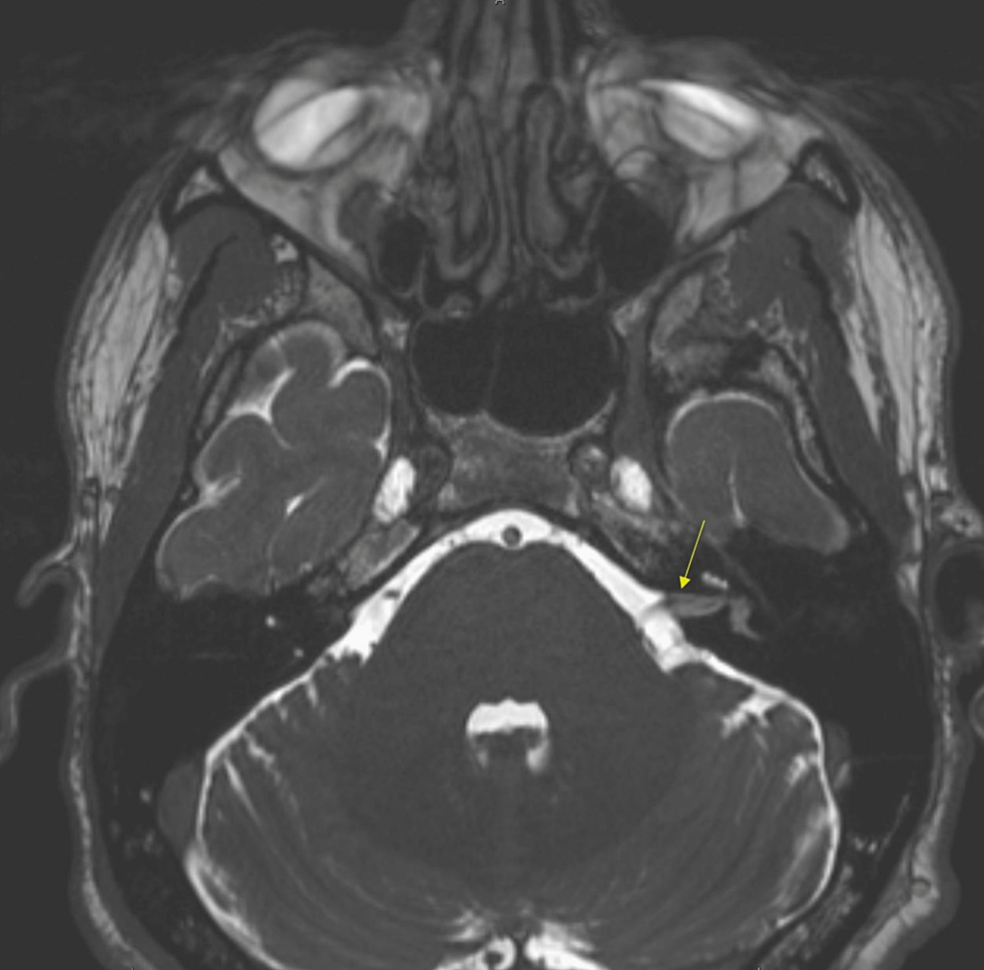
Axial T2 heavy-weighted (CISS) sequence MRI of the brain Seven months after initial imaging and three-month follow-up from the aborted gamma knife procedure. The image shows significantly decreased signal abnormality of the previously seen left IAC lesion (arrow). CISS - Constructive Interference Steady State, IAC - internal auditory canal

The patient was discussed in the lymphoma tumor board, and the team agreed to a three-month follow-up MRI with possible cerebrospinal cytology and biopsy if symptoms or image findings worsened. The most recent T1 fat-saturated sequence post-contrast MRI shows decreased enhancement of the left internal auditory canal and stable asymmetric enhancement of the left geniculate ganglion and mastoid segment of cranial nerve seven (Figures 5, 6). The patient also reported significant improvement in hearing and a near-complete resolution of his facial weakness at this last visit. The patient was deemed as improving and was scheduled to undergo yet another MRI and check-up in three months. 

## Discussion

Schwannomas are the most frequent non-cancerous neoplasm found in the internal auditory canal (IAC) and cerebellopontine angle [1]. Vestibular schwannomas, also known as acoustic neuromas, primarily develop from the vestibular portion of the vestibulocochlear nerve. These tumors originate near the vestibular ganglion close to the fundus of the IAC. Although schwannomas can arise from any cranial nerve within the IAC, including the facial nerve, typical IAC schwannomas remain confined to the canal without extending into the membranous labyrinth of the inner ear [1]. 

The presence of an acoustic neuroma is identified through the use of a contrast-enhanced MRI or CT scan. If there is any hearing loss, audiometric tests are necessary to assess the extent of hearing impairment. These IAC schwannoma lesions can also present with peripheral facial nerve palsy [1]. Schwannomas typically have a round, blunt end on MRI as well [2]. Often, there is a solid enhancing component seen in the acoustic meatus, along with a peripheral contrast cystic component in the left cerebellopontine angle [3]. These common findings were present on the patient's initial MRI (Figure 1), leading to the early diagnosis of schwannoma. Treatment of uncomplicated IAC schwannomas is often surgical excision [1].

A common differential diagnosis for schwannoma is lymphoma involving the IAC. These tumors exhibit strong enhancement on T1-weighted images accompanied by reduced signal intensities on T2-weighted images, as seen in this case (Figures 1, 2) [4]. Additionally, there is often widespread enhancement observed in the meninges both above and below the tentorium cerebelli; this, however, was not observed [4]. Patients with lymphoma also experience facial weakness, hearing loss, and dizziness [4,5]. The presence of an unrelated middle ear infection and the atypical presentation of symptoms can cause a delay in a lymphoma diagnosis [5]. High-dose steroid treatment can result in a fast improvement in symptoms, often leading to delayed diagnosis and improvement of image findings [1,4,6]. In retrospective studies looking at primary CNS lymphoma, initial steroid use led to rapid symptomatic improvement coupled with improvement in radiographic and pathology findings [6,7]. These studies also suggested biopsy rather than empirical treatment for suspected lymphoma. The patient in our case received both steroids and prophylactic antibiotics, making lymphoma hard to diagnose in this case but also a highly suspicious etiology. The patient also had continued enhancement of the geniculate ganglion, tympanic, and mastoid segments of the facial nerve on MRI, which is atypical for schwannoma, and raises inflammatory and neoplastic lesions higher on the differential [1,4,5].

Another differential diagnosis that needs to be considered in this patient is sarcoidosis. Sarcoidosis is a condition that affects multiple organs and has a diverse range of clinical presentations, often fluctuating in severity over time. The vestibulo-acoustic system is seldom impacted, with hearing loss occurring in less than 1% of patients [6]. Vestibulo-acoustic involvement often occurs simultaneously with peripheral facial nerve palsy [8]. The pathological mechanism seems to involve granulomatous meningeal inflammation in the posterior fossa and the brainstem [9,10]. Image findings are similar to those described above for the patient.

IgG4 disease needs to be considered in the differential as well. IgG4-related disease is a multisystem fibrous inflammatory disorder that affects various organs [11]. Increased MRI T2 signal intensity and decreased T1 signal intensity are often seen when involving the IAC [11]. High-dose steroid administration shows rapid improvement of symptoms but often requires continued administration [11]. The patient's current improvement of symptoms and stability, without any more steroid treatments, seems to make this differential a bit less likely.

Other inflammatory conditions, such as granulomatosis with polyangiitis, neurosyphilis, Lyme disease, leptospirosis, and metastases, may present with similar symptoms and imaging findings and should be explored, although less likely based on this patient's clinical history [12]. Investigating these would require extensive laboratory testing and patient time [12].

Although the initial suspicion of a schwannoma seemed plausible, the subsequent disappearance of the mass coupled with decreased enhancement and decreased signal abnormality of the left internal auditory canal (Figures 5, 6) gives rise to a true diagnostic challenge. The continued stable asymmetric enhancement of the left geniculate ganglion, tympanic segment, and descending mastoid segment of cranial nerve seven (Figure 5) poses the risk of a malicious or inflammatory process that won't resolve without a definitive diagnosis. The patient is clinically improving without treatment, which on its own, is a good sign and goes against the more malignant possibilities. The patient is scheduled for another three-month MRI and will be continued to be monitored for any worsening or new symptoms. As of this time, the patient has had significant improvement in hearing and a near-complete resolution of his facial weakness. The patient's tumor board and the primary team have not sought out a biopsy or invasive serological tests in light of improving symptoms and have, instead, adopted a conservative approach with careful and consistent Image monitoring. 

## Conclusions

This case report describes a diagnostic challenge in a 35-year-old patient presenting with left-sided sensorineural hearing loss and facial weakness. The initial suspicion of schwannoma was overturned by subsequent imaging findings, prompting consideration of lymphoma, sarcoidosis, IgG4 disease, or other rare inflammatory conditions. The persistence of symptoms and continued MRI findings make this case further challenging and puzzling as the patient could have lymphoma that rapidly improved or other malignant etiology without a known diagnosis or definitive treatment in place. This case, however, also demonstrates the role of conservative management in patients with complex presentations. Ultimately, the patient will need definitive tissue or cerebrospinal fluid analysis to fully assess for lymphoma, IgG4, or sarcoidosis if he fails to improve or if image findings worsen at any of his subsequent monitoring visits. Having this diagnostic data would allow for full prognostication and allow for complete management by the tumor board or the patient's primary team. 
